# Changes in distributions of waist circumference, waist-to-hip ratio and waist-to-height ratio over an 18-year period among Chinese adults: a longitudinal study using quantile regression

**DOI:** 10.1186/s12889-019-6927-6

**Published:** 2019-06-06

**Authors:** Xiwen Qian, Chang Su, Bing Zhang, Guoyou Qin, Huijun Wang, Zhenyu Wu

**Affiliations:** 1 Department of Biostatistics, School of Public Health, Key Laboratory of Public Health Safety and Collaborative Innovation Center of Social Risks Governance in Health, Fudan University, Shanghai, 200032 People’s Republic of China; 2 National Institute for Nutrition and Health, Chinese Center for Disease Control and Prevention, 29 Nanwei Road, Xicheng District, Beijing, 100050 People’s Republic of China

**Keywords:** Waist circumference, Waist-to-hip ratio, Waist-to-height ratio, Abdominal obesity, Quantile regression

## Abstract

**Background:**

Little is known about the long-term shifts in distributions of three abdominal-obesity-related indicators, waist circumference (WC), waist-to-hip ratio (WHpR) and waist-to-height ratio (WHtR) among Chinese adults. Traditional mean regression models used in the previous analyses were limited in their ability to capture cross-distribution among effects. The current study aims to describe the shift in distribution of WC, WHpR, and WHtR over a period of 18 years (1993–2011) in China, and to reveal quantile-specific associations of the three indicators with key covariates.

**Methods:**

Longitudinal data from seven waves of the China Health and Nutrition Surveys (CHNS) in 1993, 1997, 2000, 2004, 2006, 2009 and 2011 were analyzed. The LMS method was used to illustrate the gender-specific quantile curves of WC, WHtR and WHpR over age. Separate gender-stratified longitudinal quantile regressions were employed to investigate the effect of important factors on the trends of the three indicators.

**Results:**

A total of 11,923 participants aged 18–65 years with 49,507 observations were included in the analysis. The density curves of WC, WHtR and WHpR shifted to right and became wider. The three outcomes all increased with age and increased more at upper percentiles. From the multivariate quantile regression, physical activity was negatively associated in both genders; smoking only had a negative effect on male indicators. Education and drinking behavior both had opposite effects on the three indicators between men and women. Marital status and income were positively associated with the shifts in WC, WHtR and WHpR in male and female WC, while urbanicity index had a positive effect on three outcomes in men but inconsistent effect among female outcomes.

**Conclusions:**

The abdominal-obesity related indicators of the Chinese adults experienced rapid growth according to our population-based, age- and gender-specific analyses. Over the 18-year study period, major increases in WC, WHtR and WHpR were observed among Chinese adults. Specifically, these increases were greater at upper percentiles and in men. Age, physical activity, energy intake, drinking, smoking, education, income and urbanicity index were associated with elevated abdominal obesity indicators, and the effects differed among percentiles and between genders*.*

**Electronic supplementary material:**

The online version of this article (10.1186/s12889-019-6927-6) contains supplementary material, which is available to authorized users.

## Background

Obesity has become a serious problem that threatens public health. Body mass index (BMI), which is defined as weight divided by the square of the height (kg/m^2^), is probably the most commonly used index to evaluate overall body fatness and to determine overweight or obesity in adults. However, one major drawback of BMI is that it fails to consider the distribution of fat throughout the body. Abdominal fat, i.e., that around the heart, liver and kidneys, has been found by no means to be less pathogenic than general obesity, but rather has more significant relationship with heavy disease burden [1–3].

Abdominal obesity can usually be evaluated by the three most popularly used indicators: waist circumference (WC), waist-to-hip ratio (WHpR) and waist-to-height ratio (WHtR). Significant increases in these indicators have been reported in developed countries. An Australian 12-year cohort study reported increases of 4.32 cm and 6.25 cm in WC from 1999 to 2011 for men and women, respectively [4]. The prevalence of WC over 102 cm among men aged 40–79 years has increased by 13.1% in northeast European cities from 2003 to 2010 [5]. In the US, the overall age adjusted mean WC increased 3 cm from 1999 to 2012 [6]. Developing countries such as China have also experienced a serious obesity crisis. The prevalence of abdominal obesity thus increased dramatically from 17.3 to 39.4% between 1997 and 2009 [3]. The age-adjusted prevalence of abdominal obesity in China was 35.3% in men and 51.7% in women in 2011 [7].

More importantly, accumulating evidence has shown the positive correlation of abdominal obesity indicators with the risk of chronic diseases. For example, WC alone or WC combined with BMI is more predictive than BMI alone for hypertension [8] and obesity-related mortality [9]. WHtR is a better predictor of metabolic syndrome [10], diabetes, hypertension and cardiovascular disease (CVD) [11, 12]. WHpR is thought to more precisely measure visceral fat because it attenuates the influence of subcutaneous fat by considering hip circumference (HC) [13, 14], which is also inversely connected to dyslipidemia, diabetes and CVD [15]. Although the optimal predictive abdominal obesity indicators for chronic diseases varied among studies, what remains certain is that these three indicators are negatively associated with various diseases.

The past decades witnessed great development in China. The popularizing of mass education and the process of urbanization had a dramatic influence on people’s health. The health status of Chinese population was challenged by changes in dietary pattern and lifestyle. Studies also reported the important association between socioeconomic status and obesity [16, 17]. Therefore, socioeconomic and lifestyle factors were considered in our study, to further explore the association of these variables and changes of abdominal obesity related measures.

Traditionally, a general linear regression model was frequently performed to study the effect of obesity-related covariates on the conditional mean of the dependent variable. However, this method is not suitable if the effect of explanatory variables differs at different levels of the outcome, and cannot make full use of the overall distribution. By contrast, quantile regression builds an array of equations that are regressed to defined quantiles without extra hypotheses in distribution. Thus, it is more robust against outliers or skewness to the response variable than is ordinary linear regression [18]. Meanwhile, quantile regression can provide a detailed description of the association between covariates and each quantile of the response variable.

In the current study, we aim to describe the secular shift of abdominal obesity in adults, as depicted by WC, WHtR and WHpR, and to explore the relationships between covariates and changes of indicators at each quantile. Our results provide new perspectives on the population health and may encourage researchers and policy makers to control, prevent and decrease the epidemic of abdominal obesity.

## Methods

### Study population

Data for this study was derived from the China Health and Nutrition Surveys (CHNS), a large-scale longitudinal survey designed to cover key public health risk factors and health outcomes and, demographic, socioeconomic factors at the individual, household and community levels. The CHNS aimed to examine the effects of social and economic change across time on public health. The sample of this large-scale survey was selected randomly from eight provinces in the first wave in 1989. Within each province, stratified sampling was used to select cities and counties. In later survey years, more provinces were involved, and more data was collected. Detailed information is available in the profile [19, 20].

In the current study, adults aged 18 to 65 from the seven latest waves in 1993, 1997, 2000, 2004, 2006, 2009 and 2011 were analyzed. Due to the longitudinal nature of our study, only subjects with more than one records were considered as qualified participants. We excluded subjects without measurements of WC, height or HC and participants whose WC was outside the range of 45 to 150 cm, whose HC was outside the range of 55 to 150 cm, or whose height was outside the range of 120 to 200 cm were also excluded as extreme outliers. Missing values were imputed with multiple imputation method. A total of 11,923 individuals with 49,507 records were involved in our final analysis.

### Outcomes

The outcomes of interest were WC, WHtR and WHpR. WC, HC and height were collected by physical measurements methods. WC was taken at a midpoint between the bottom of the rib cage and the top of the iliac crest at the end of exhalation. HC was taken at the level of maximal gluteal protrusion. Both WC and HC were measured using a SECA tape to the nearest 0.1 cm. Height was measured without shoes to the nearest 0.2 cm using a portable stadiometer. WHtR and WHpR were computed as WC divided by height and HC, respectively.

### Covariates

Categorical covariates included sex (male, female), educational level (none or primary school, middle school, senior school or above), smoking status (no, yes), drinking status (no, yes) and marital status (unmarried, married, divorced and other). Continuous covariates included age, energy intake, total physical activity, per capita family annual income, and urbanicity level.

Data of smoking, drinking, energy intake, physical activity, educational level, marital status and income was collected by questionnaires and dietary survey. Variables were defined as follows:

#### Energy intake

average daily energy intake for each individual, calculated based on the data of detailed food consumption during three consecutive days at both the household and individual level.

#### Physical activities (PA)

indicated by average metabolic equivalents of task (MET) hours per day in a week, estimated from four aspects: occupational, domestic, active leisure and travel. The MET is defined as the ratio of a person’s working metabolic rate (and therefore the rate of energy consumption) to his or her basal metabolic rate. One MET is defined as 1 kcal/kg-hour of energy cost.

#### Per capita income

average individual income, calculated based on reported gross annual household income and was inflated to 2011 values using the Consumer Price Index [21] and categorized into year-specific tertiles.

#### Urbanicity index

A total score at the community level to describe the characteristics and degree of urbanization, calculated by a multicomponent continuous scale developed specifically for the CHNS [22]. Each community was evaluated by 12 components with a maximum of 10 points of each, including economic activity, traditional markets, modern markets, population density, transportation infrastructure, communications, sanitation, health infrastructure, housing, education, diversity and social services. This variable was categorized into tertiles in the regression models.

### Statistical analysis

The demographic, socioeconomic and lifestyle features in each wave were described in Table 1. Continuous variables were expressed as medians, the first quartile (25th) and the third quartile (75th). Categorical variables were presented with frequency and percentage. We used trend Chi-square test for categorical variables and Kruskal–Wallis test for continuous variables to examine the difference over time.

**Table 1:** Demographic characteristics of the study sample (*N* = 49,507)

	1993	1997	2000	2004	2006	2009	2011	P*
Sample size	5442	7546	7686	7571	7465	7363	6434	
Male (%)	2638 (48)	3801 (50)	3703 (48)	3660 (48)	3581 (48)	3542 (48)	3056 (47)	
Female (%)	2804 (52)	3745 (50)	3983 (52)	3911 (52)	3884 (52)	3821 (52)	3378 (53)	
Age (years)	38.62 (30.00,46.54)	40.20 (30.56,48.82)	42.44 (33.05,50.85)	45.14 (35.83,53.25)	46.26 (37.47,54.37)	46.94 (38.86,55.61)	48.62 (40.77,56.86)	< 0.001
Smoke								< 0.001
No (%)	3528 (65)	4978 (66)	5180 (67)	5052 (67)	5061 (68)	4997 (68)	4423 (69)	
Yes (%)	1914 (35)	2565 (34)	2502 (33)	2516 (33)	2403 (32)	2366 (32)	2011 (31)	
Drink								< 0.001
No (%)	3406 (63)	4647 (62)	4883 (64)	4943 (65)	4907 (66)	4722 (64)	4170 (65)	
Yes (%)	2036 (37)	2889 (38)	2788 (36)	2626 (35)	2557 (34)	2641 (36)	2264 (35)	
Energy intake (1000 kcal/d)	2.373 (1.959,2.836)	2.428 (2.019,2.929)	2.326 (1.913,2.797)	2.258 (1.841,2.747)	2.290 (1.845,2.821)	2.163 (1.779,2.622)	2.053 (1.637,2.557)	< 0.001
Total physical activity(100METs/d)	2.11 (1.00,3.37)	2.60 (1.28,4.56)	2.07 (1.03,4.00)	1.73 (0.80,3.46)	1.72 (0.76,3.33)	1.75 (0.84,3.28)	1.85 (0.90,3.25)	< 0.001
Education level								< 0.001
None/primary (%)	2902 (53)	3692 (49)	3321 (43)	2974 (39)	2778 (37)	2678 (36)	2279 (35)	
Middle school (%)	1651 (30)	2369 (31)	2575 (34)	2665 (35)	2592 (35)	2754 (37)	2422 (38)	
Senior/above (%)	888 (16)	1472 (20)	1782 (23)	1930 (25)	2093 (28)	1931 (26)	1733 (27)	
Marital status								< 0.001
Unmarried (%)	625 (11.5)	965 (12.8)	809 (10.5)	582 (7.7)	466 (6.2)	375 (5.1)	267 (4.1)	
Married (%)	4682 (86.0)	6318 (83.7)	6578 (85.6)	6655 (87.9)	6668 (89.3)	6581 (89.4)	5779 (89.8)	
Divorced (%)	21 (0.4)	46 (0.6)	68 (0.9)	92 (1.2)	93 (1.2)	128 (1.7)	138 (2.1)	
Other (%)	114 (2.1)	217 (2.9)	231 (3.0)	242 (3.2)	238 (3.2)	279 (3.8)	250 (3.9)	
Individual income (Yuan)	2595.48 (1502.49,4392.02)	3564.56 (2055.24,5714.71)	4432.20 (2355.11,7263.67)	5261.33 (2693.60,9672.23)	6024.26 (2872.05,10,962.58)	8889.46 (4832.47,15,685.91)	11,036.93 (5860.76,18,708.16)	< 0.001
Urbanicity index	43.94 (32.75,60.88)	51.21 (35.18,66.90)	55.19 (42.69,76.76)	58.74 (44.27,81.11)	61.85 (47.26,82.31)	64.09 (51.07,84.69)	67.99 (51.63,86.24)	< 0.001
WC (cm)	74.0 (70.0,80.0)	77.0 (71.0,83.2)	78.0 (72.0,85.0)	80.0 (74.0,87.0)	80.0 (74.0,87.5)	82.0 (75.0,89.2)	83.0 (76.0,90.4)	< 0.001
Height (cm)	160.0 (154.1166.0)	160.4 (154.9166.8)	160.8 (155.0,167.2)	161.0 (155.3167.5)	161.2 (156.0,168.0)	161.5 (156.0,168.0)	161.7 (156.0,168.0)	< 0.001
HC (cm)	89.0 (85.0,94.0)	91.0 (86.6,96.0)	93.0 (88.0,98.0)	93.0 (88.0,98.0)	93.1 (89.0,98.2)	94.1 (89.5,99.8)	95.0 (90.0,100.0)	< 0.001

Continuous variables are expressed as medians (25th percentile, 75th percentile), categorical variables are expressed as frequency (percentages %)

* Chi-square tests for categorical variables and Kruskal–Wallis tests for continuous variables

Abbreviation: WC, waist circumference; HC, hip circumference

To illustrate the shifts of WC, WHtR and WHpR between 1993 and 2011, kernel density plots were used to display distributions. To illustrate the age-specific smoothed quantile curves for these three indicators, LMS (lambda, mu, and sigma) quantile regression were constructed, in which the parameters *λ*, *μ* and *σ* were chosen to maximize a penalized log-likelihood in the VGAM package in R version 3.3.2 (R Development Core Team, Vienna, Austria).

Finally, gender-stratified longitudinal analyses were conducted to investigate the time trend of abdominal obesity measures and influencing factors. Due to limited space, we multiplied both WHtR and WHpR by 100 to avoid the regression coefficients close to zero. Longitudinal quantile regression models with fixed effects for each outcome of interest were built in three steps using the *lqmm* package: Model 1 only included year, and the coefficient measured the crude yearly change of the outcome; Model 2 adjusted individual-level features, including age, energy intake, PA, smoking status and drinking status, educational level, marital status and per capita annual income, so the coefficient reflected the yearly change conditional on individual-level covariates; Model 3 controlled a community-level urbanicity index based on Model 2. Thus, in Model 3, the coefficient of time indicated the effect of time-varying factors or unavailable or unmeasurable covariates like culture, environment and social policy, after controlling individual level and community level covariates. A quadratic term of age was included in Model 2 and Model 3.

## Results

The individual-, household-, and community-level characteristics of the studied samples are presented in Table 1. Approximately one-third of the participants reported smoking or drinking history in each round of the survey. The daily energy intake and total PA showed a decreased trend. WC, the fundamental predictor of abdominal obesity, increased by almost 10 cm, whereas the median HC and height increased 6 cm and 1.6 cm, respectively, over an 18-year period.

### Trends of the distribution changes in WC, WHtR and WHpR from 1993 to 2011

**Fig. 1:**
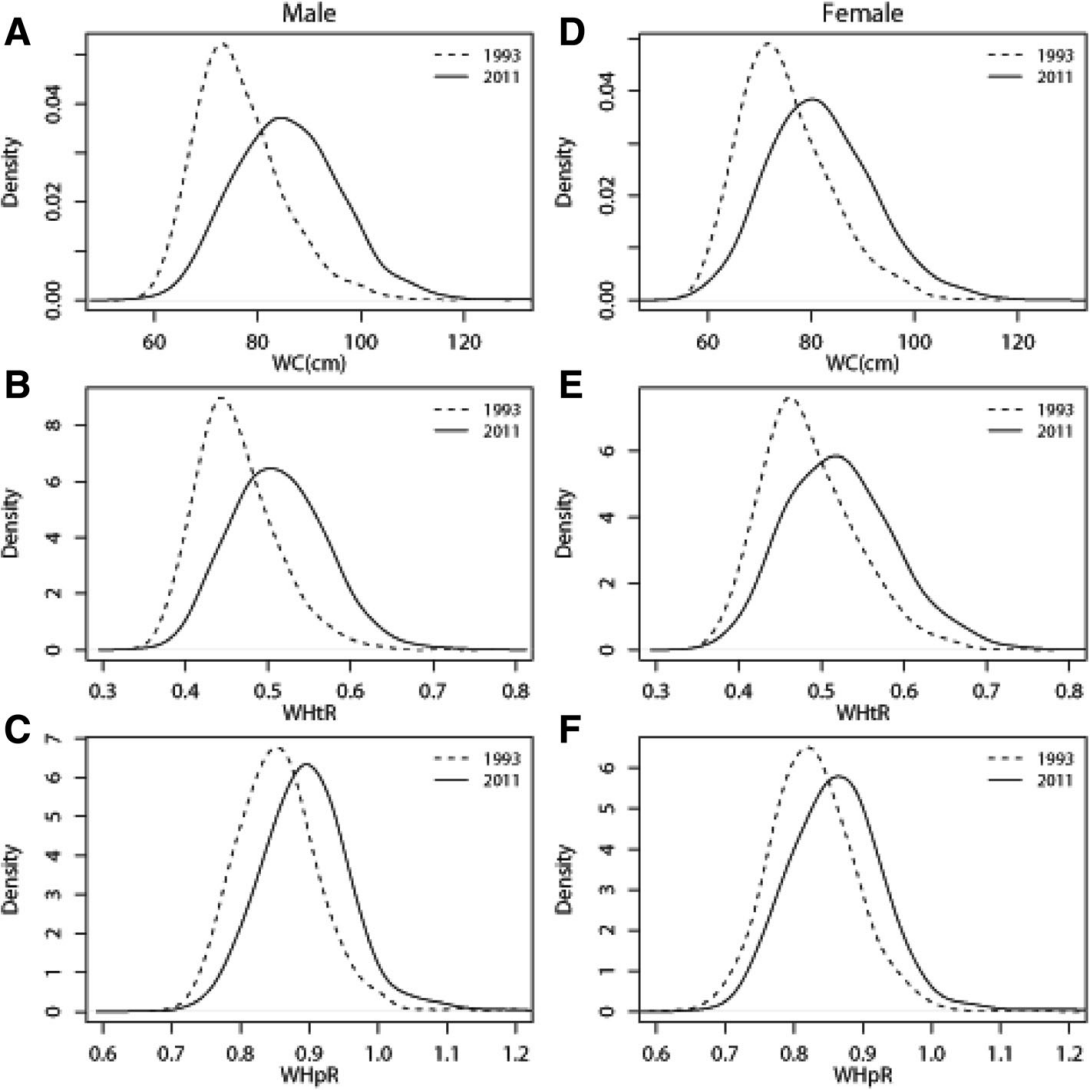
Shifts in distributions of WC, WHtR and WHpR for Chinese adults between 1993 and 2011

For both genders, from 1993 to 2011, the density curves all shifted to right and became wider, which meant that the proportion of subjects with high WC, WHtR and WHpR increased with time (Fig. 1; in Additional file 1: Figure S1). However, greater increases in WC, WHtR and WHpR were found in men than in women.

### Shifts in the quartile curves of WC, WHtR and WHpR

**Fig. 2:**
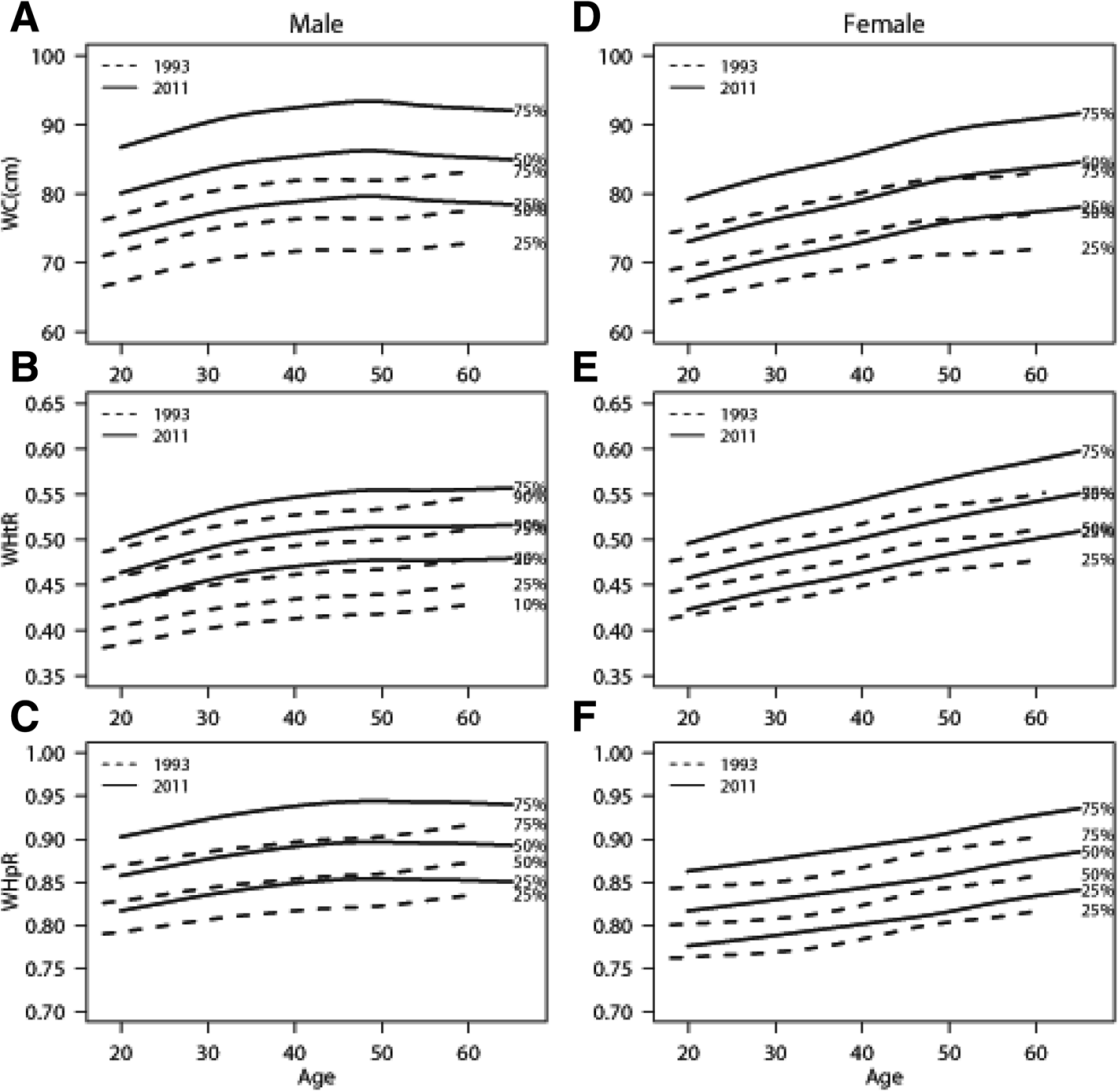
Quantile curves by age of WC, WHtR and WHpR for adults in 1993 and 2011

Percentile curves, using the LMS method, for both genders in each age group were shown in selected years (Fig. 2; in Additional file 1: Figure S1- S4). All the solid lines (2011) were above the dotted lines (1993) (Fig. 2), which is in consistence with the results in Fig. 1 showing that people were getting fatter in 2011. Specifically, for the quartile levels of WC, WHtR increased dramatically with age, while WHpR was relatively stable. Note that WC, WHtR and WHpR increased almost linearly in women. In men, the curves were not as smooth and became flat or even decreased with age after 50 years of age.

### Gender-specific quantile regression results for each outcome

Time effects, which were estimated by the yearly coefficients in three quantile models using WC, WHtR or WHpR as an outcome, are listed in Table 2. Model 1 suggested a significant increase in all the outcomes of interest from 10th percentile to the 90th percentile for both sexes. WC increased more at upper percentiles while WHpR increased more at lower percentiles. For example, WC increased 1.400 cm (95% CI: 1.286, 1.514) and 1.227 cm (95% CI: 1.091, 1.360) per year at 10th percentile in men and women, respectively; at 90th percentile, WC increased 1.507 cm (95% CI: 1.327, 1.686) and 1.337 cm (95% CI: 1.209, 1.460) in men and women. After adjusting for lifestyle and socioeconomic variables in Model 2, the time effect on the three outcomes declined but still remained significant (Table 2). When the urbanicity index was considered in Model 3, the time effect on male WHtR, WHpR and female WC became slightly stronger compared with Model 2, while on other declined at all percentiles.

**Table 2:** Yearly coefficients (standard error) in quantile regressions using WC, WHtR or WHpR as outcomes

	10th	25th	50th	75th	90th
Male					
**WC**					
Model 1	1.400^a^ (0.057)	1.279^a^ (0.070)	1.337^a^ (0.035)	1.440^a^ (0.041)	1.507^a^ (0.089)
Model 2	1.107^a^ (0.032)	1.110^a^ (0.034)	1.133^a^ (0.032)	1.184^a^ (0.033)	1.176^a^ (0.032)
Model 3	1.102^a^ (0.042)	1.114^a^ (0.038)	1.152^a^ (0.039)	1.160^a^ (0.045)	1.204^a^ (0.039)
WHtR					
Model 1	0.798^a^ (0.021)	0.750^a^ (0.024)	0.795^a^ (0.023)	0.800^a^ (0.024)	0.750^a^ (0.034)
Model 2	0.535^a^ (0.023)	0.543^a^ (0.023)	0.552^a^ (0.022)	0.558^a^ (0.022)	0.572^a^ (0.023)
Model 3	0.546^a^ (0.022)	0.551^a^ (0.021)	0.560^a^ (0.020)	0.567^a^ (0.020)	0.579^a^ (0.021)
WHpR					
Model 1	0.714^a^ (0.037)	0.671^a^ (0.022)	0.650^a^ (0.028)	0.664^a^ (0.022)	0.632^a^ (0.041)
Model 2	0.461^a^ (0.027)	0.476^a^ (0.029)	0.487^a^ (0.028)	0.495^a^ (0.029)	0.512^a^ (0.028)
Model 3	0.471^a^ (0.026)	0.475^a^ (0.023)	0.491^a^ (0.023)	0.511^a^ (0.022)	0.531^a^ (0.024)
Female					
WC					
Model 1	1.227^a^ (0.068)	1.001^a^ (0.029)	1.272^a^ (0.029)	1.332^a^ (0.058)	1.337^a^ (0.063)
Model 2	0.751^a^ (0.044)	0.763^a^ (0.042)	0.782^a^ (0.043)	0.791^a^ (0.046)	0.808^a^ (0.047)
Model 3	0.753^a^ (0.032)	0.786^a^ (0.032)	0.788^a^ (0.032)	0.801^a^ (0.031)	0.818^a^ (0.035)
WHtR					
Model 1	0.749^a^ (0.029)	0.747^a^ (0.026)	0.752^a^ (0.026)	0.769^a^ (0.029)	0.802^a^ (0.030)
Model 2	0.361^a^ (0.025)	0.368 (0.024)	0.374^a^ (0.024)	0.379^a^ (0.024)	0.390^a^ (0.024)
Model 3	0.356^a^ (0.025)	0.365^a^ (0.025)	0.362^a^ (0.026)	0.377^a^ (0.026)	0.390^a^ (0.026)
WHpR					
Model 1	0.750^a^ (0.032)	0.701^a^ (0.022)	0.700^a^ (0.027)	0.707^a^ (0.034)	0.696^a^ (0.048)
Model 2	0.437^a^ (0.026)	0.442^a^ (0.026)	0.456^a^ (0.026)	0.463^a^ (0.025)	0.477^a^ (0.026)
Model 3	0.407^a^ (0.023)	0.421^a^ (0.025)	0.434^a^ (0.023)	0.444^a^ (0.022)	0.450^a^ (0.025)

Model 1 includes year only; Model 2 includes year, age, education, energy intake, physical activity, income, smoking and drinking history; Model 3 includes all the components of Model 2 and the urbanicity index

^a^*P* < 0.001

Abbreviation: WC, waist circumference; WHtR, waist-to-height ratio; WHpR, waist-to-hip ratio

Detailed information of Model 3 is provided in (Additional file 1 :Table S1-S3). Among the three outcomes that all increased with age and increased more at upper percentiles, the increases in WC, male WHtR and male WHpR were at a decreasing speed as the coefficients of quadratic age were significantly negative. Results show that the estimates from least squared model are different from quantile regression.

Physical activities had a significant negative association -- which was much stronger at lower percentiles than that at upper percentiles -- with WC, WHtR and WHpR in both men and women. For example, female WC decreased 0.305 cm (95% CI: -0.385, − 0.225) and 0.236 cm (95% CI: -0.335, − 0.137) with an additional 100 MET-hour of physical activity per day at 10th and 90th percentiles, respectively; female 100-fold WHtR and WHpR decreased 0.145 (95% CI: -0.187, − 0.103) and 0.167 (95% CI: -0.217, − 0.115) respectively at 10th percentile, and 0.105 (95% CI: -0.151, − 0.059) and 0.124 (95% CI: -0.183, − 0.066) respectively at 90th percentile, with an additional 10,000 MET-hour of physical activity per day. Smoking only significantly associated with male WC and WHtR. WC in male smokers tended to be 1.061 cm (95% CI: -1.470, − 0.652) smaller than that in nonsmokers at 10th percentile, and 1.044 cm (95% CI: -1.453, − 0.635) at 90th percentile. WHtR in male smokers was also smaller, and the negative effect of smoking was stronger at lower percentiles.

Interestingly, drinking and educational level had completely opposite effects on the three outcomes between sexes. Drinking caused a significant increase in all percentiles of WC, WHtR and WHpR among men. While female non-drinkers with media value of WC were prone to have 0.599 cm (95% CI: -1.091, − 0.107) larger WC, as well as larger WHtR and WHpR, but significance was only shown in WC. Higher educational level tended to cause larger WC and WHtR in men, but smaller WC, WHtR and WHpR in women. The effects of education at upper percentiles were stronger than at lower percentiles in men, but weaker in women. For instance, men receiving senior schooling or above had approximately 1.775 cm (95% CI: 1.221, 2.329) more of WC at 10th percentiles and 1.790 cm (95% CI: 1.235, 2.344) at 90th percentile, compared with those with no or primary schooling. For women with senior schooling or above, WC at 10th percentile was 2.257 cm (95% CI: -2.816, − 1.698) and at 90th percentile was 2.250 cm (95% CI: -2.809, − 1.690) smaller than those least-educated.

Compared with never-married men and women, the married had notably larger outcomes, and the effects of marriage on three indicators were stronger in men than in women. WC in married men and women were around 2.116 cm (95% CI at 50th percentile: 1.452, 2.780) and 1.651 cm (95% CI at 50th percentile: 0.959, 2.342), respectively larger than that in unmarried men and women. Higher level of income was positively associated with three indicators in men, but the association was inconsistent among three outcomes in women. For example, men with relatively high income had 1.913 cm (95% CI: 1.561, 2.266) lager WC at 10th percentiles and 1.926 cm (95% CI: 1.574, 2.278) at 90th percentile. In women, high level of income contributed to an increase by 0.603 cm (95% CI at 50th percentile: 0.243, 0.963) in WC but a decrease in WHpR. For men, urbanicity index had a positive effect which was stronger at upper percentiles on the WC, WHtR and WHpR. Living in a community with high urbanicity index resulted in an increment of 1.651 cm (95% CI: 1.577, 2.743) in WC at 10th percentile in men and 1.667 cm (95% CI: 1.127, 2.207) at 90th percentile, compared with men living in a community with low urbanicity index. Similar results were also found in women for WC, but not WHtR and WHpR.

## Discussion

To the best of our knowledge, the current study is the first age- and gender-specific analysis in a Chinese population to explore the secular trends of three indicators of abdominal obesity and their relation with potential risk factors. We found the three indicators increased more in men and at upper percentiles over a period of 18 years.

Previous studies using CHNS data showed that WC and the prevalence of abdominal obesity had increased greatly from 1993 to 2009 in both sexes [23], especially among those living in rural regions and among individuals aged 40–59 years [24]. Similar trends for WC and WHpR were found in US adults [6, 25] and the Finnish population [26]. Studies on the change of WHtR and WHpR are limited. We believe that WHtR and WHpR are important for evaluating abdominal obesity. WHtR adjusts for height, which tends to decrease with age and differs among ethnic groups and regions, and WHpR distinguishes “pear-shaped” from “apple-shaped” body types. According to our analyses, WC, WHtR and WHpR have increased rapidly over the past decades, but the patterns of age-specific increase are different. WHtR behaved similarly to WC because there was little change in population height over the decades. The WHpR curves were relatively flat, which means that the WHpR was not as dramatically increased as the other two indicators. The reason for this phenomenon may be that the increase of WC is usually accompanied by an increase in HC, which makes their ratio more stable. Therefore, WHpR may not be appropriate to describe the secular trend of abdominal obesity, though it is a valuable prognostic indicator for many chronic diseases.

Gender-related differences in the shifting patterns of the three indicators can be summarized from two aspects: on the one hand, the extent of increment is greater in men. For example, with WC, the percentile curves in 2011 were much higher than those in 1993 in men, but the extent of the increase in women was not as remarkable as that in men. On the other hand, the age-specific increase is different. Again, taking WC as an example, percentile curves for men started to decrease after 50 years of age, but the curves were still climbing in women. It has been confirmed that gender is an important factor influencing body composition and the accumulation and distribution of body fat due to the effect of sex hormones [27]; it may also be due to different lifestyles.

In the multivariate quantile regression analyses, the opposite effects of education level on the indicators among genders have been noted. Such a divergence has also been reported in other population [4, 28]. A systematic review concluded from numerous related studies that the effect of education on obesity usually depends on the country’s level of development and on a modification by gender [17]. It remains possible that in China, women with a high level of education are more likely to live a healthy life because of their knowledge of good health practices [28]; thus, they may have lower abdominal obesity indicators. However, this is different for men, as highly educated men may have busier work schedules, more business trips, more social engagements and business dinners, leading to more energy intake and less time for physical activities. Speaking of income, probably both men and women with high income have busier work thus pay less attention to exercise and healthy diet. Our finding about the association between urbanicity and abdominal obesity indicators was consistent with other studies from China [29] and other developing countries like India [30]. A high level of community urbanicity means good access to food, especially fast food, heavy use of motorized transportation and less open space, which may help explain the increasing abdominal obesity indicators.

The positive association between marital status and obesity or abdominal obesity has been reported [31, 32]. It can be explained by the fact that single people have stronger intention of weight loss to stay attractive while married people tend to have less time for physical activity. Physical activity is an acknowledged protective factor for abdominal obesity because it is a major determinant of energy consumption [25, 33, 34] and is negatively associated with high WC, BMI and WHpR [28]. In our study, we found that physical activity is especially meaningful in men and those with low values of abdominal obesity indicators. The divergent effect of drinking on the indicators between genders may also partly relate to education, income and employment. Highly educated women with better jobs and greater socializing skills may need to drink occasionally and are more likely to pay particular attention to their appearance [35]. In fact, studies on the association between alcohol consumption and abdominal obesity have presented inconsistent findings for either or both genders [36–39], which may be because alcohol is metabolized differently in men and women. However, the insight mechanism has not been well elucidated [35]. With respect to smoking, it generally had a significant negative association with the WC and WHtR in men. Divergent effects of smoking were also discussed in other literatures. One Chinese-population-based study demonstrated that regular smoking was associated with increased WC and WHtR after adjusting for BMI [40], while others claimed a negative association between smoking and body weight [41] or a positive association between smoking and increase in abdominal fat [34, 42]. The mechanisms through which smoking affects body weight and fat distribution are complex and still not fully understood. The effects of cigarette smoking on body weight are probably mediated by the smoking behavior which may serve as a behavioral alternative to eating, resulting in decreased food intake [41]. Besides, like many anti-obesity drugs, nicotine as a sympathomimetic agent, can reduce body weight by increasing energy expenditure [43].

Our study had several limitations. Detailed information regarding eating, drinking and smoking behaviors, as well as variables like occupation were not included in our database. Limitations also stem from the sample attrition. However, the rate of lost to follow-up was low relative to the large sample size and data was collected from fixed regions, so we assumed the missing data were uninformative. Sensitive analyses with imputed data were also conducted, providing results that were similar to those of our study. Despite these limitations, our study has several strengths. The longitudinal nature of variables at the individual, household and community levels was considered, and the effects on changes were analyzed in a comprehensive way. Moreover, the use of quantile regression allowed a more detailed and robust investigation of the distributions and trends of the indicators.

## Conclusions

Over the 18-year study period, rapid increases in abdominal-obesity related measurements, WC, WHtR and WHpR were observed among Chinese adults. Specifically, these increases were greater at upper percentiles and greater in men. Age, physical activity, energy intake, drinking, smoking, educational level, income level and community-level urbanicity were associated with elevated abdominal obesity indicators, and the effects differed among percentiles and between genders. Regardless of which indicator is used, the remarkable increase of abdominal obesity in China is worthy of great attention. Furthermore, the increase is very likely to continue after 2011 accompanied with the ongoing processes of urbanicity and modernization. Therefore, effective strategies for preventing and controlling the epidemic of abdominal obesity are needed to diminish the negative effects on public health.

## Additional file

Additional file 1: This file contains 4 figures and 3 tables that present additional results obtained in the study that we consider important to publish. **Figure S1.** depicts the shifts in distributions of WC, WHtR and WHpR for Chinese adults in each wave. **Figure S2-S4.** show the quantile curves by age of WC, WHtR and WHpR respectively for adults in each wave. **Table S1-S3.** provide the coefficients and standard errors from multivariate quantile regression for 10th, 25th, 50th, 75th and 90th percentiles of WC, WHtR and WHpR, respectively. (DOCX 11252 kb)

## Abbreviations

BMI: Body mass index
CHNS: China Health and Nutrition Surveys
HC: Hip circumference
WC: Waist circumference
WHpR: Waist-to hip ratio
WHtR: Waist-to-height ratio
