# Supplementary material for: Changes in distributions of waist circumference, waist-to-hip ratio and waist-to-height ratio over an 18-year period among Chinese adults: a longitudinal study using quantile regression
Source: BMC Public Health. 2019 Jun 6;19:700. doi: 10.1186/s12889-019-6927-6 (PMC6555739; doi:10.1186/s12889-019-6927-6)
Supplement: Supplementary file 1 — This file contains 4 figures and 3 tables that present additional results obtained in the study that we consider important to publish. Figure S1. depicts the shifts in distributions of WC, WHtR and WHpR for Chinese adults in each wave. Figure S2-S4. show the quantile curves by age of WC, WHtR and WHpR respectively for adults in each wave. Table S1-S3. provide the coefficients and standard errors from multivariate quantile regression for 10th, 25th, 50th, 75th and 90th percentiles of WC, WHtR and WHpR, respectively. (DOCX 11252 kb) [file 12889_2019_6927_MOESM1_ESM.docx]

**Supplementary materials**

Figure S1. Shifts in distributions of WC, WHtR and WHpR for Chinese adults in each wave.


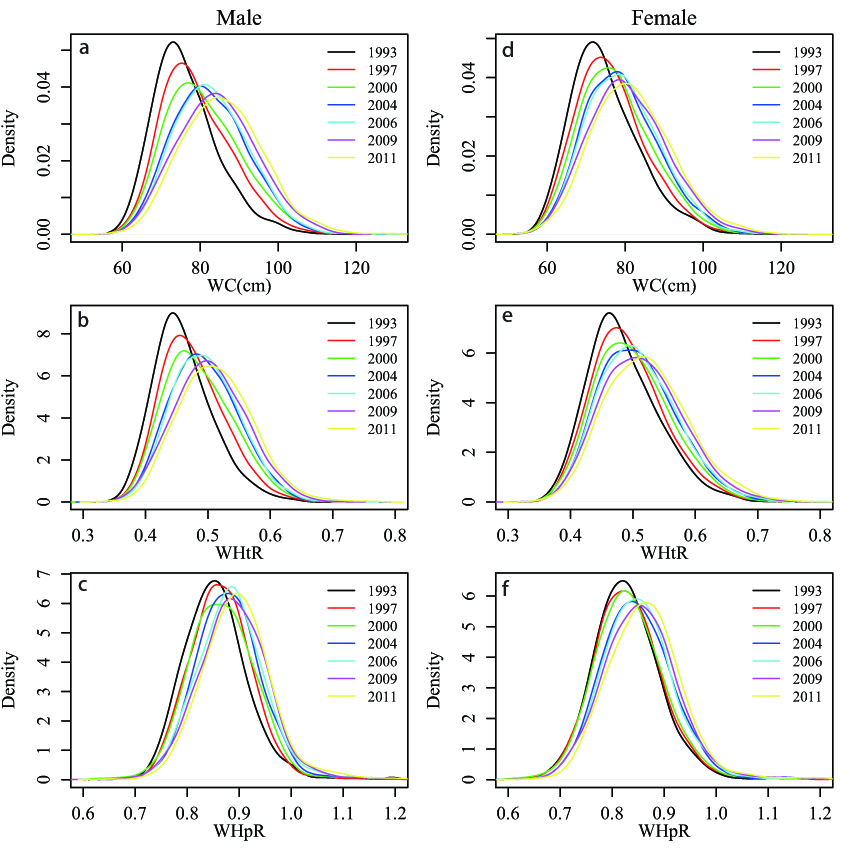


Figure S2. Quantile curves by age of WC for adults in each wave.


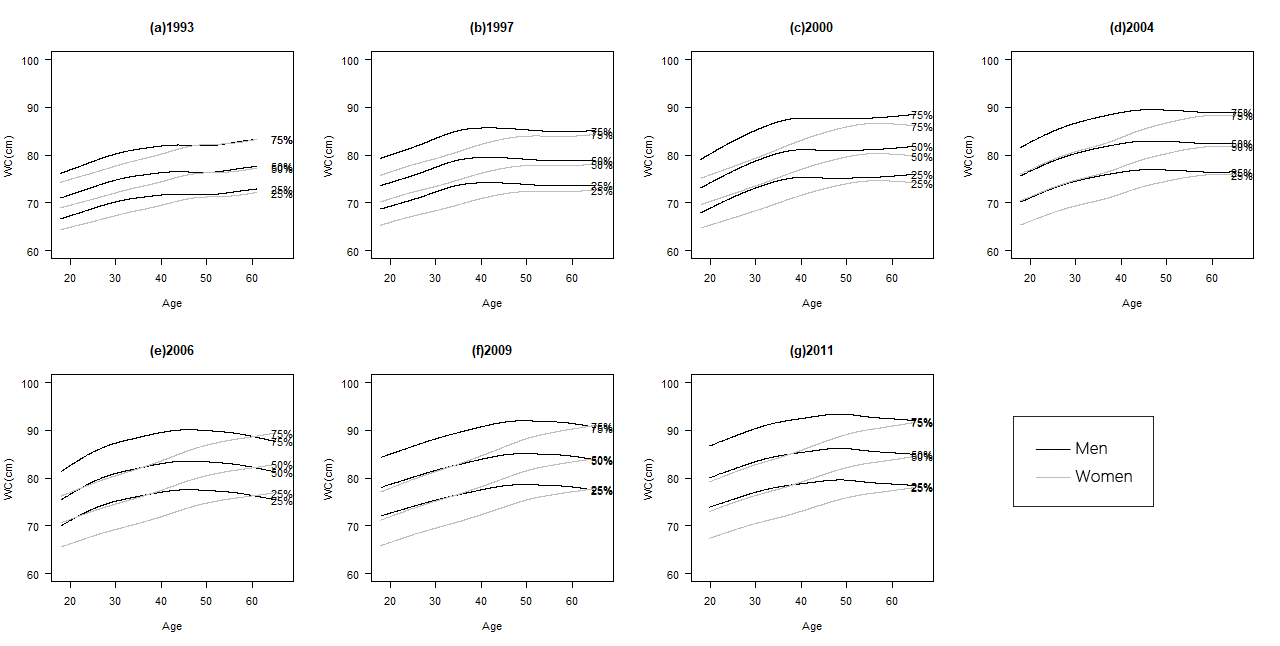


Figure S3. Quantile curves by age of WHtR for adults in each wave.


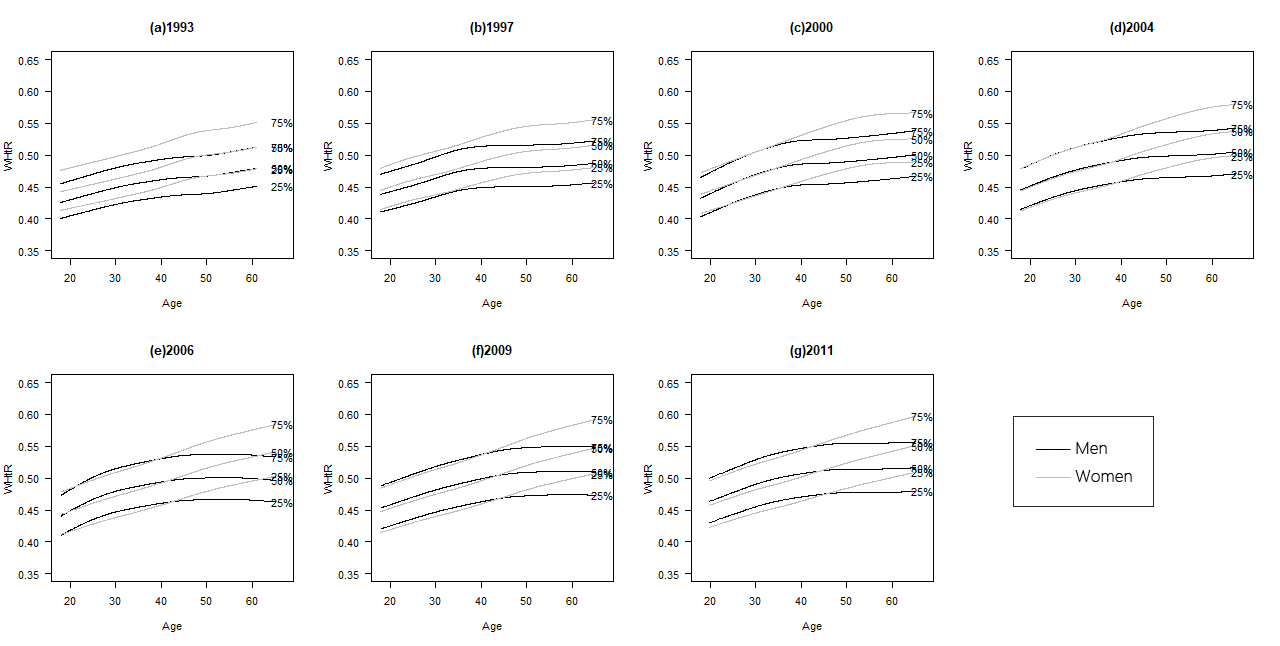


Figure S4. Quantile curves by age of WHpR for adults in each wave.


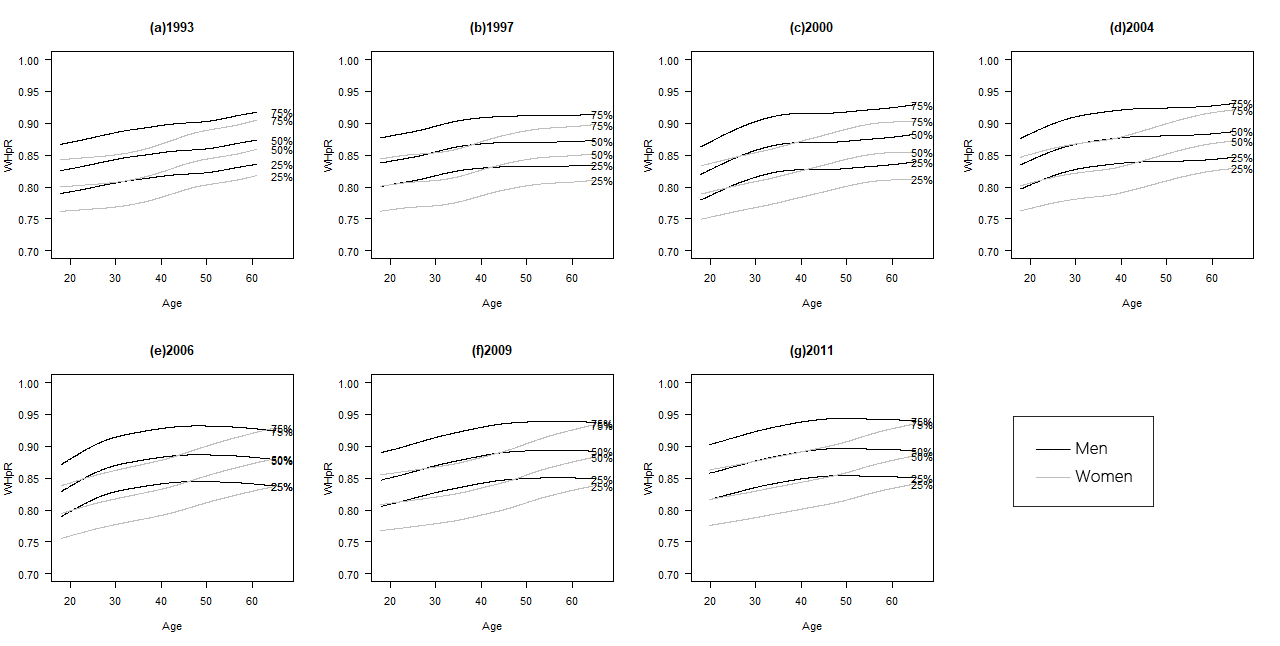


Table S1: Coefficient (standard error) from quantile regression for 10^th^, 25^th^, 50^th^, 75^th^ and 90^th^ percentiles of WC in Model 3.

|  | 10^th^ | 25^th^ | 50^th^ | 75^th^ | 90^th^ | Mean |
| --- | --- | --- | --- | --- | --- | --- |
| **Male** |  |  |  |  |  |  |
| Intercept | 62.150^a^ (0.973) | 62.152^a^ (0.975) | 62.168^a^ (0.974) | 62.171^a^ (0.974) | 62.188^a^ (0.974) | 62.842^a^ (0.704) |
| Year | 1.102^a^ (0.042) | 1.114^a^ (0.038) | 1.152^a^ (0.039) | 1.160^a^ (0.045) | 1.204^a^ (0.039) | 1.030^a^ (0.031) |
| Age | 0.183 (0.098) | 0.210^b^ (0.060) | 0.513^a^ (0.049) | 0.569^a^ (0.103) | 0.905^a^ (0.084) | 0.565^a^ (0.035) |
| Age*Age | -0.002 (0.002) | -0.001 (0.001) | -0.005^a^ (0.001) | -0.004 (0.002) | -0.008^a^ (0.001) | -0.005^a^ (0.0004) |
| Energy intake (1000 kcal/d) | 0.161 (0.091) | 0.165 (0.093) | 0.206^c^ (0.091) | 0.213^c^ (0.088) | 0.259^c^ (0.095) | 0.022 (0.055) |
| Physical activity (100MET/d) | -0.446^a^ (0.034) | -0.453^a^ (0.035) | -0.422^a^ (0.034) | -0.415^a^ (0.037) | -0.373^a^ (0.039) | -0.098^a^ (0.023) |
| Nonsmoker | Ref | Ref | Ref | Ref | Ref | Ref |
| Smoker | -1.061^a^ (0.200) | -1.061^a^ (0.199) | -1.054^a^ (0.200) | -1.052^a^ (0.201) | -1.044^a^ (0.200) | -0.668^a^ (0.113) |
| Nondrinker | Ref | Ref | Ref | Ref | Ref | Ref |
| Drinker | 0.985^a^ (0.153) | 0.985^a^ (0.155) | 0.995^a^ (0.154) | 0.997^a^ (0.154) | 1.006^a^ (0.154) | 0.331^a^ (0.101) |
| Educational level |  |  |  |  |  |  |
| None/primary | Ref | Ref | Ref | Ref | Ref | Ref |
| Middle school | 1.226^a^ (0.243) | 1.228^a^ (0.243) | 1.238^a^ (0.243) | 1.239^a^ (0.243) | 1.249^a^ (0.243) | 0.971^a^ (0.157) |
| Senior/above | 1.775^a^ (0.271) | 1.776^a^ (0.271) | 1.782^a^ (0.271) | 1.783^a^ (0.270) | 1.790^a^ (0.271) | 1.394^a^ (0.204) |
| Marital status |  |  |  |  |  |  |
| Unmarried | Ref | Ref | Ref | Ref | Ref | Ref |
| Married | 2.107^a^ (0.325) | 2.107^a^ (0.325) | 2.116^a^ (0.325) | 2.118^a^ (0.324) | 2.127^a^ (0.325) | 0.646^b^ (0.198) |
| Divorced | 1.516 (0.851) | 1.516 (0.851) | 1.517 (0.851) | 1.517 (0.851) | 1.517 (0.851) | 0.515 (0.461) |
| Others | 0.969 (0.668) | 0.969 (0.668) | 0.969 (0.668) | 0.969 (0.668) | 0.968 (0.668) | -0.327 (0.427) |
| Income level |  |  |  |  |  |  |
| Low | Ref | Ref | Ref | Ref | Ref | Ref |
| Middle | 0.367^c^ (0.173) | 0.366^c^ (0.172) | 0.372^c^ (0.173) | 0.373^c^ (0.173) | 0.380^c^ (0.172) | 0.219^c^ (0.103) |
| High | 1.913^a^ (0.172) | 1.915^a^ (0.172) | 1.920^a^ (0.172) | 1.921^a^ (0.172) | 1.926^a^ (0.172) | 0.712^a^ (0.117) |
| Urbanicity level |  |  |  |  |  |  |
| Low | Ref | Ref | Ref | Ref | Ref | Ref |
| Middle | 0.572^c^ (0.240) | 0.573^c^ (0.240) | 0.579^c^ (0.240) | 0.580^c^ (0.240) | 0.586^c^ (0.240) | 0.545^a^ (0.134) |
| High | 1.651^a^ (0.265) | 1.654^a^ (0.265) | 1.660^a^ (0.264) | 1.661^a^ (0.264) | 1.667^a^ (0.264) | 1.579^a^ (0.182) |
| **Female** |  |  |  |  |  |  |
| Intercept | 61.832^a^ (1.138) | 61.845^a^ (1.14) | 61.845^a^ (1.139) | 61.851^a^ (1.139) | 61.859^a^ (1.138) | 63.533^a^ (0.741) |
| Year | 0.753^a^ (0.032) | 0.786^a^ (0.032) | 0.788^a^ (0.032) | 0.801^a^ (0.031) | 0.818^a^ (0.035) | 0.711^a^ (0.031) |
| Age | 0.152 (0.078) | 0.388^a^ (0.062) | 0.403^a^ (0.065) | 0.512^a^ (0.087) | 0.676^a^ (0.120) | 0.321^a^ (0.035) |
| Age*Age | -0.001 (0.001) | -0.004^a^ (0.001) | -0.002^b^ (0.001) | -0.002 (0.001) | -0.003 (0.002) | -0.001^b^ (0.0003) |
| Energy intake (1000 kcal/d) | 0.227^c^ (0.100) | 0.253^c^ (0.100) | 0.255^c^ (0.100) | 0.266^c^ (0.097) | 0.285^b^ (0.101) | 0.193^b^ (0.070) |
| Physical activity (100MET/d) | -0.305^a^ (0.039) | -0.282^a^ (0.041) | -0.280^a^ (0.039) | -0.262^a^ (0.041) | -0.236^a^ (0.048) | -0.103^a^ (0.022) |
| Nonsmoker | Ref | Ref | Ref | Ref | Ref | Ref |
| Smoker | 0.225 (0.607) | 0.225 (0.607) | 0.225 (0.607) | 0.226 (0.607) | 0.226 (0.607) | 0.050 (0.309) |
| Nondrinker | Ref | Ref | Ref | Ref | Ref | Ref |
| Drinker | -0.600^c^ (0.240) | -0.599^c^ (0.240) | -0.599^b^ (0.241) | -0.598^c^ (0.241) | -0.598^c^ (0.241) | -0.444^b^ (0.151) |
| Educational level |  |  |  |  |  |  |
| None/primary | Ref | Ref | Ref | Ref | Ref | Ref |
| Middle school | -0.622^b^ (0.222) | -0.615^b^ (0.222) | -0.614^c^ (0.223) | -0.612^c^ (0.223) | -0.608^c^ (0.223) | -0.434^b^ (0.160) |
| Senior/above | -2.257^a^ (0.273) | -2.253^a^ (0.273) | -2.253^a^ (0.274) | -2.252^a^ (0.274) | -2.250^a^ (0.274) | -1.314^a^ (0.217) |
| Marital status |  |  |  |  |  |  |
| Unmarried | Ref | Ref | Ref | Ref | Ref | Ref |
| Married | 1.641^a^ (0.338) | 1.650^a^ (0.339) | 1.651^a^ (0.338) | 1.655^a^ (0.338) | 1.661^a^ (0.338) | 1.253^a^ (0.276) |
| Divorced | 0.682 (0.861) | 0.682 (0.861) | 0.682 (0.861) | 0.682 (0.861) | 0.682 (0.861) | 0.515 (0.561) |
| Others | 0.302 (0.594) | 0.302 (0.594) | 0.302 (0.594) | 0.301 (0.594) | 0.301 (0.594) | 0.515 (0.382) |
| Income level |  |  |  |  |  |  |
| Low | Ref | Ref | Ref | Ref | Ref | Ref |
| Middle | 0.059 (0.128) | 0.063 (0.128) | 0.064 (0.128) | 0.066 (0.128) | 0.069 (0.128) | -0.042 (0.102) |
| High | 0.599^b^ (0.176) | 0.602^b^ (0.176) | 0.603^b^ (0.176) | 0.604^b^ (0.176) | 0.605^b^ (0.176) | 0.059^a^ (0.117) |
| Urbanicity level |  |  |  |  |  |  |
| Low | Ref | Ref | Ref | Ref | Ref | Ref |
| Middle | 0.415 (0.203) | 0.419 (0.202) | 0.420 (0.203) | 0.422 (0.203) | 0.425 (0.202) | 0.156 (0.133) |
| High | 0.313^c^ (0.284) | 0.318^c^ (0.284) | 0.318^c^ (0.284) | 0.319^c^ (0.284) | 0.321^c^ (0.284) | 0.445 (0.181) |

^a^: *P*< 0.001, ^b^: *P*<0.01, ^c^: *P*<0.05.

Table S2: Coefficient (standard error) from quantile regression for 10^th^, 25^th^, 50^th^, 75^th^ and 90^th^ percentiles of WHtR*100 in Model 3.

|  | 10^th^ | 25^th^ | 50^th^ | 75^th^ | 90^th^ | Mean |
| --- | --- | --- | --- | --- | --- | --- |
| **Male** |  |  |  |  |  |  |
| Intercept | 37.146^a^ (0.603) | 37.148^a^ (0.604) | 37.152^a^ (0.604) | 37.154^a^ (0.604) | 37.16^a^ (0.604) | 37.676^a^ (0.414) |
| Year | 0.546^a^ (0.022) | 0.551^a^ (0.021) | 0.560^a^ (0.020) | 0.567^a^ (0.020) | 0.579^a^ (0.021) | 0.508^a^ (0.018) |
| Age | 0.229^a^ (0.060) | 0.262^a^ (0.040) | 0.336^a^ (0.029) | 0.390^a^ (0.036) | 0.494^a^ (0.035) | 0.332^a^ (0.021) |
| Age*Age | -0.003^b^ (0.001) | -0.003^a^ (0.001) | -0.003^a^ (0.0004) | -0.003^a^ (0.001) | -0.004^a^ (0.001) | -0.003^a^ (0.0002) |
| Energy intake (1000 kcal/d) | 0.091 (0.046) | 0.095 (0.047) | 0.105^c^ (0.047) | 0.112^c^ (0.046) | 0.127^c^ (0.047) | 0.015 (0.033) |
| Physical activity (100MET/d) | -0.213^a^ (0.019) | -0.211^a^ (0.019) | -0.204^a^ (0.019) | -0.197^a^ (0.019) | -0.183^a^ (0.019) | -0.047^a^ (0.014) |
| Nonsmoker | Ref | Ref | Ref | Ref | Ref | Ref |
| Smoker | -0.656^a^ (0.098) | -0.655^a^ (0.098) | -0.653^a^ (0.098) | -0.652^a^ (0.098) | -0.649^a^ (0.098) | -0.400^a^ (0.067) |
| Nondrinker | Ref | Ref | Ref | Ref | Ref | Ref |
| Drinker | 0.471^a^ (0.081) | 0.472^a^ (0.080) | 0.475^a^ (0.081) | 0.476^a^ (0.081) | 0.479^a^ (0.081) | 0.164^b^ (0.06) |
| Educational level |  |  |  |  |  |  |
| None/primary | Ref | Ref | Ref | Ref | Ref | Ref |
| Middle school | 0.378^c^ (0.167) | 0.379^c^ (0.167) | 0.382^c^ (0.167) | 0.383^c^ (0.167) | 0.386^c^ (0.167) | 0.379^a^ (0.092) |
| Senior/above | 0.429^c^ (0.169) | 0.430^c^ (0.169) | 0.431^c^ (0.169) | 0.432^c^ (0.169) | 0.434^c^ (0.169) | 0.410^a^ (0.119) |
| Marital status |  |  |  |  |  |  |
| Unmarried | Ref | Ref | Ref | Ref | Ref | Ref |
| Married | 0.953^a^ (0.156) | 0.954^a^ (0.157) | 0.956^a^ (0.157) | 0.957^a^ (0.156) | 0.960^a^ (0.157) | 0.372^b^ (0.118) |
| Divorced | 0.749^c^ (0.337) | 0.749^c^ (0.337) | 0.749^c^ (0.337) | 0.749^c^ (0.337) | 0.749^c^ (0.337) | 0.344 (0.274) |
| Others | 0.390 (0.325) | 0.390 (0.325) | 0.390 (0.325) | 0.390 (0.325) | 0.390 (0.325) | -0.210 (0.253) |
| Income level |  |  |  |  |  |  |
| Low | Ref | Ref | Ref | Ref | Ref | Ref |
| Middle | 0.085 (0.084) | 0.085 (0.083) | 0.087 (0.084) | 0.088 (0.084) | 0.090 (0.084) | 0.105 (0.061) |
| High | 0.717^a^ (0.115) | 0.718^a^ (0.115) | 0.720^a^ (0.115) | 0.721^a^ (0.115) | 0.722^a^ (0.115) | 0.330^a^ (0.07) |
| Urbanicity level |  |  |  |  |  |  |
| Low |  |  |  |  |  |  |
| Middle | 0.304^c^ (0.122) | 0.305^c^ (0.122) | 0.306^c^ (0.122) | 0.307^c^ (0.122) | 0.309^c^ (0.122) | 0.205^b^ (0.079) |
| High | 0.643^a^ (0.135) | 0.644^a^ (0.135) | 0.645^a^ (0.135) | 0.646^a^ (0.135) | 0.648^a^ (0.135) | 0.618^a^ (0.106) |
| **Female** |  |  |  |  |  |  |
| Intercept | 40.633^a^ (0.692) | 40.637^a^ (0.692) | 40.636^a^ (0.691) | 40.642^a^ (0.691) | 40.649^a^ (0.692) | 41.356^a^ (0.474) |
| Year | 0.356^a^ (0.025) | 0.365^a^ (0.025) | 0.362^a^ (0.026) | 0.377^a^ (0.026) | 0.390^a^ (0.026) | 0.335^a^ (0.020) |
| Age | 0.089^c^ (0.034) | 0.155^a^ (0.038) | 0.143^a^ (0.032) | 0.266^a^ (0.044) | 0.388^a^ (0.07) | 0.170^a^ (0.022) |
| Age*Age | 0.0003 (0.001) | -0.001 (0.001) | 0.001 (0.002) | 0.0002 (0.001) | -0.001 (0.001) | 0.0002 (0.003) |
| Energy intake (1000 kcal/d) | 0.161^c^ (0.063) | 0.169^c^ (0.062) | 0.167^c^ (0.063) | 0.180^b^ (0.063) | 0.195^b^ (0.063) | 0.116^c^ (0.045) |
| Physical activity (100MET/d) | -0.145^a^ (0.021) | -0.139^a^ (0.02) | -0.142^a^ (0.021) | -0.124^a^ (0.022) | -0.105^a^ (0.023) | -0.047^b^ (0.014) |
| Nonsmoker | Ref | Ref | Ref | Ref | Ref | Ref |
| Smoker | -0.406 (0.313) | -0.406 (0.313) | -0.406 (0.313) | -0.405 (0.313) | -0.405 (0.313) | -0.152 (0.198) |
| Nondrinker | Ref | Ref | Ref | Ref | Ref | Ref |
| Drinker | -0.299 (0.154) | -0.299 (0.154) | -0.299 (0.154) | -0.298 (0.154) | -0.298 (0.154) | -0.267^b^ (0.097) |
| Educational level |  |  |  |  |  |  |
| None/primary | Ref | Ref | Ref | Ref | Ref | Ref |
| Middle school | -0.825^a^ (0.159) | -0.823^a^ (0.159) | -0.824^a^ (0.159) | -0.820^a^ (0.159) | -0.817^a^ (0.159) | -0.511^a^ (0.102) |
| Senior/above | -2.149^a^ (0.168) | -2.148^a^ (0.168) | -2.148^a^ (0.168) | -2.147^a^ (0.168) | -2.145^a^ (0.168) | -1.359^a^ (0.139) |
| Marital status |  |  |  |  |  |  |
| Unmarried | Ref | Ref | Ref | Ref | Ref | Ref |
| Married | 0.761 (0.161) | 0.764 (0.161) | 0.763^a^ (0.161) | 0.768^a^ (0.161) | 0.773^a^ (0.161) | 0.771^a^ (0.177) |
| Divorced | 0.044 (0.647) | 0.044 (0.647) | 0.044 (0.647) | 0.044 (0.647) | 0.045 (0.647) | 0.250 (0.360) |
| Others | 0.227 (0.439) | 0.227 (0.439) | 0.227 (0.439) | 0.227 (0.439) | 0.227 (0.439) | 0.443 (0.245) |
| Income level |  |  |  |  |  |  |
| Low | Ref | Ref | Ref | Ref | Ref | Ref |
| Middle | -0.108 (0.115) | -0.107 (0.115) | -0.107 (0.115) | -0.105 (0.115) | -0.103 (0.115) | -0.059 (0.066) |
| High | -0.036 (0.130) | -0.035 (0.130) | -0.036 (0.130) | -0.034 (0.130) | -0.032 (0.130) | -0.067 (0.075) |
| Urbanicity level |  |  |  |  |  |  |
| Low | Ref | Ref | Ref | Ref | Ref | Ref |
| Middle | 0.200 (0.146) | 0.201 (0.146) | 0.200 (0.146) | 0.203 (0.146) | 0.206 (0.146) | -0.009 (0.086) |
| High | -0.012 (0.189) | -0.010 (0.189) | -0.010 (0.189) | -0.009 (0.189) | -0.007 (0.189) | -0.024 (0.115) |

^a^: *P*< 0.001, ^b^: *P*<0.01, ^c^: *P*<0.05.

Table S3: Coefficient (standard error) from quantile regression for 10^th^, 25^th^, 50^th^, 75^th^ and 90^th^ percentiles of WHpR*100 in Model 3.

|  | 10^th^ | 25^th^ | 50^th^ | 75^th^ | 90^th^ | Mean |
| --- | --- | --- | --- | --- | --- | --- |
| **Male** |  |  |  |  |  |  |
| Intercept | 78.063^a^ (0.627) | 78.065^a^ (0.626) | 78.073^a^ (0.626) | 78.083^a^ (0.626) | 78.093^a^ (0.626) | 78.134^a^ (0.577) |
| Year | 0.471^a^ (0.026) | 0.475^a^ (0.023) | 0.491^a^ (0.023) | 0.511^a^ (0.022) | 0.531^a^ (0.024) | 0.467^a^ (0.023) |
| Age | 0.106^a^ (0.068) | 0.132^a^ (0.065) | 0.284^a^ (0.029) | 0.471^c^ (0.054) | 0.650 (0.060) | 0.287^a^ (0.030) |
| Age*Age | -0.002^a^ (0.001) | -0.001^a^ (0.001) | -0.003^a^ (0.0004) | -0.005 (0.001) | -0.007 (0.001) | -0.002^a^ (0.003) |
| Energy intake (1000 kcal/d) | 0.005 (0.061) | 0.010 (0.059) | 0.030 (0.058) | 0.055 (0.060) | 0.080 (0.057) | 0.020 (0.050) |
| Physical activity (100MET/d) | -0.236^a^ (0.025) | -0.235^a^ (0.026) | -0.217^a^ (0.025) | -0.196^a^ (0.024) | -0.171 (0.026) | -0.111^a^ (0.020) |
| Nonsmoker | Ref | Ref | Ref | Ref | Ref | Ref |
| Smoker | -0.204 (0.115) | -0.203 (0.115) | -0.199 (0.115) | -0.194 (0.115) | -0.189 (0.116) | -0.207^c^ (0.096) |
| Nondrinker | Ref | Ref | Ref | Ref | Ref | Ref |
| Drinker | 0.432^a^ (0.095) | 0.433^a^ (0.096) | 0.438^a^ (0.095) | 0.443^a^ (0.095) | 0.448^a^ (0.096) | 0.164 (0.089) |
| Educational level |  |  |  |  |  |  |
| None/primary | Ref | Ref | Ref | Ref | Ref | Ref |
| Middle school | 0.254^c^ (0.131) | 0.255 (0.131) | 0.259 (0.131) | 0.265 (0.131) | 0.271 (0.131) | 0.265^c^ (0.124) |
| Senior/above | 0.174 (0.146) | 0.175 (0.146) | 0.177 (0.146) | 0.181 (0.146) | 0.184 (0.147) | 0.291 (0.153) |
| Marital status |  |  |  |  |  |  |
| Unmarried | Ref | Ref | Ref | Ref | Ref | Ref |
| Married | 0.886^a^ (0.195) | 0.886^a^ (0.195) | 0.891^a^ (0.195) | 0.896^a^ (0.195) | 0.900^a^ (0.195) | 0.610^a^ (0.172) |
| Divorced | 1.395^c^ (0.562) | 1.395^c^ (0.562) | 1.395^c^ (0.562) | 1.396^c^ (0.562) | 1.396^c^ (0.562) | 1.070 (0.399) |
| Others | 0.285 (0.429) | 0.285 (0.429) | 0.285 (0.429) | 0.285 (0.429) | 0.285 (0.429) | 0.033 (0.367) |
| Income level |  |  |  |  |  |  |
| Low | Ref | Ref | Ref | Ref | Ref | Ref |
| Middle | -0.042 (0.095) | -0.042 (0.095) | -0.039 (0.095) | -0.036 (0.095) | -0.032 (0.095) | 0.044 (0.094) |
| High | 0.440^a^ (0.113) | 0.440^a^ (0.112) | 0.443^a^ (0.113) | 0.446^a^ (0.113) | 0.449^a^ (0.112) | 0.297^b^ (0.104) |
| Urbanicity level |  |  |  |  |  |  |
| Low | Ref | Ref | Ref | Ref | Ref | Ref |
| Middle | 0.209 (0.140) | 0.210 (0.140) | 0.213 (0.140) | 0.215 (0.140) | 0.218 (0.140) | 0.119 (0.112) |
| High | 0.329^c^ (0.145) | 0.331^c^ (0.146) | 0.333^c^ (0.145) | 0.338^c^ (0.145) | 0.342^c^ (0.146) | 0.372^b^ (0.140) |
| **Female** |  |  |  |  |  |  |
| Intercept | 79.188^a^ (0.863) | 79.194^a^ (0.863) | 79.200^a^ (0.863) | 79.205^a^ (0.863) | 79.208^a^ (0.863) | 79.390^a^ (0.630) |
| Year | 0.407^a^ (0.023) | 0.421^a^ (0.025) | 0.434^a^ (0.023) | 0.444^a^ (0.022) | 0.450^a^ (0.025) | 0.417^a^ (0.025) |
| Age | -0.184 (0.047) | -0.077 (0.068) | 0.033^c^ (0.039) | 0.140^c^ (0.065) | 0.188^c^ (0.084) | 0.018 (0.031) |
| Age*Age | 0.003 (0.001) | 0.002 (0.001) | 0.001 (0.0003) | 0.0005 (0.001) | 0.001 (0.002) | 0.001^a^ (0.0003) |
| Energy intake (1000 kcal/d) | 0.298 (0.076) | 0.311^a^ (0.077) | 0.323^a^ (0.076) | 0.335^a^ (0.077) | 0.341^a^ (0.075) | 0.261^a^ (0.067) |
| Physical activity (100MET/d) | -0.167^a^ (0.025) | -0.157^a^ (0.025) | -0.144^a^ (0.025) | -0.128^a^ (0.026) | -0.124^a^ (0.029) | -0.117^a^ (0.021) |
| Nonsmoker | Ref | Ref | Ref | Ref | Ref | Ref |
| Smoker | -0.275 (0.328) | -0.275 (0.327) | -0.275 (0.328) | -0.275 (0.328) | -0.275 (0.328) | -0.118 (0.265) |
| Nondrinker | Ref | Ref | Ref | Ref | Ref | Ref |
| Drinker | -0.309 (0.157) | -0.309 (0.157) | -0.308 (0.157) | -0.308 (0.157) | -0.308 (0.157) | -0.316^c^ (0.142) |
| Educational level |  |  |  |  |  |  |
| None/primary | Ref | Ref | Ref | Ref | Ref | Ref |
| Middle school | -0.808^a^ (0.129) | -0.805^a^ (0.128) | -0.802^a^ (0.129) | -0.799^a^ (0.129) | -0.798^a^ (0.129) | -0.640^a^ (0.131) |
| Senior/above | -1.423^a^ (0.145) | -1.421^a^ (0.145) | -1.419^a^ (0.145) | -1.417^a^ (0.145) | -1.416^a^ (0.145) | -1.187^a^ (0.167) |
| Marital status |  |  |  |  |  |  |
| Unmarried | Ref | Ref | Ref | Ref | Ref | Ref |
| Married | 0.722^b^ (0.218) | 0.726^b^ (0.218) | 0.731^b^ (0.217) | 0.735^b^ (0.217) | 0.737^b^ (0.219) | 0.797^a^ (0.236) |
| Divorced | -0.482 (0.632) | -0.482 (0.632) | -0.482 (0.632) | -0.482 (0.632) | -0.482 (0.632) | -0.172 (0.496) |
| Others | 0.582 (0.356) | 0.582 (0.356) | 0.582 (0.356) | 0.582 (0.356) | 0.582 (0.356) | 0.632 (0.329) |
| Income level |  |  |  |  |  |  |
| Low | Ref | Ref | Ref | Ref | Ref | Ref |
| Middle | -0.189 (0.126) | -0.188 (0.126) | -0.186 (0.126) | -0.184 (0.126) | -0.183 (0.126) | -0.089 (0.099) |
| High | -0.192^c^ (0.109) | -0.190^c^ (0.109) | -0.188 (0.109) | -0.187 (0.108) | -0.186 (0.109) | -0.127 (0.110) |
| Urbanicity level |  |  |  |  |  |  |
| Low | Ref | Ref | Ref | Ref | Ref | Ref |
| Middle | 0.063 (0.160) | 0.065 (0.16) | 0.067 (0.161) | 0.069 (0.161) | 0.070 (0.161) | -0.019 (0.117) |
| High | -0.812^a^ (0.166) | -0.810^a^ (0.166) | -0.807^a^ (0.166) | -0.806^a^ (0.166) | -0.804^a^ (0.166) | -0.730^a^ (0.146) |

^a^: *P*< 0.001, ^b^: *P*<0.01, ^c^: *P*<0.05.
